# Acute heart failure caused by a giant hepatocellular metastatic tumor of the right atrium

**DOI:** 10.1186/1749-8090-6-102

**Published:** 2011-08-26

**Authors:** Panagiotis Dedeilias, Ioannis Nenekidis, Ioannis Koukis, Vania Anagnostakou, Niki Paparizou, Spyros Zompolos, Efstratios Apostolakis

**Affiliations:** 1 1st Department of Cardiac Surgery, Evangelismos General Hospital, Athens, Greece; 2 Department of Cardiothoracic Surgery, 401 Army General Hospital, Athens, Greece; 3 Radiology Department, Evangelismos General Hospital, Athens, Greece; 4 Anaesthesiology Department, Evangelismos General Hospital, Athens, Greece; 5 Cardiology Department, Kalamata General Hospital, Kalamata, Greece; 6 Cardiothoracic Department, University Hospital of Ioannina, Ioannina, Greece

## Abstract

We present a symptomatic 40-year-old cirrhotic man who presented with sudden onsets of syncope. Echocardiography revealed right ventricular outflow track obstruction caused by a huge right atrial mass. The tumor was surgically excised under cardiopulmonary bypass. Although no primary cancerous lesion in the liver was detected, histopathology revealed that the mass was a metastatic hepatocellular carcinoma. The aim of this report is to show the value of urgent preoperative computed tomography and its contribution in the operative strategy. The importance of urgent surgical treatment with tricuspid valve sparing tumor resection is emphasized even though the prognosis for such patients is dismal. We also discuss the further management options of such rare cases

## Background

Hepatocellular metastatic carcinomas to the heart are uncommon malignant tumors that are usually located to the right atrium. Prompt diagnosis of their presence is of major clinical importance because although rare they can cause obstructive phenomena, heart failure and even sudden cardiac death [1-3]. Herein, we present a patient with a metastatic hepatocellular carcinoma located in the right atrium and invading the right ventricle, the pre op workout and the subsequent management

### Case report

**Figure 1:**
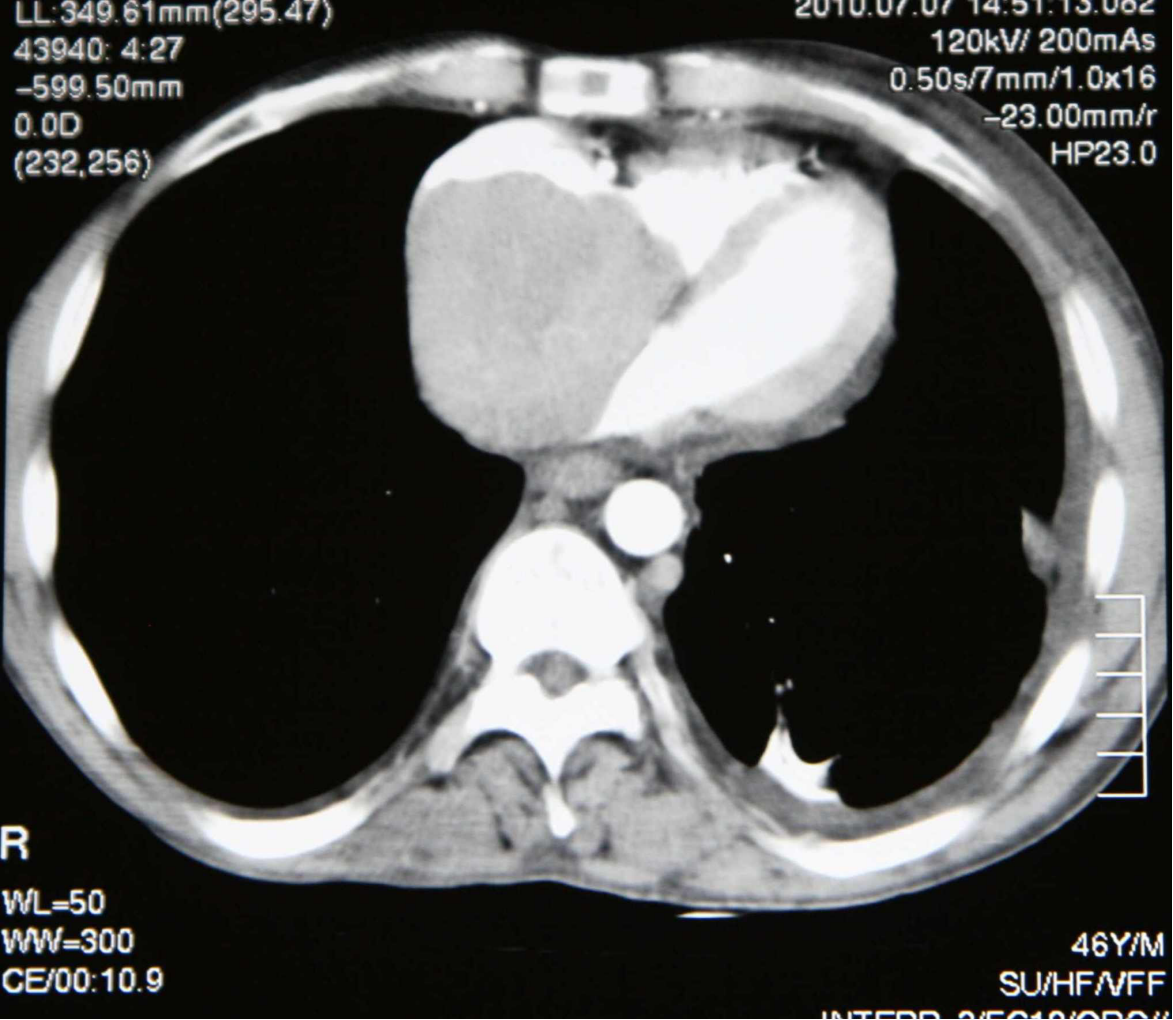
**CT angiogram verifying the presence of a mass inside the right atrium occupying almost the whole cavity**.

A 40 year old cirrhotic male was admitted to the cardiology emergency department due to sudden onsets of syncope. He also presented with exertional dyspnoea accompanied by continuous chest pain and cough. His medical history included hepatitis B marker positive. Clinical examination revealed cyanotic and swollen head and neck with distended jugular veins up to the angle of the mandible. His blood pressure was 98/62 mmHg and the oxygen saturation on room air was 90%. Cardiac rhythm was normal but the rate was increased. Electrocardiogram (ECG) showed sinus tachycardia (145 beats/min). Urgent cardiac ultrasound revealed a giant mass that partially occupied the right atrium. A subsequent urgent chest CT angiography presented a huge non-homogenous tumour occupying almost the entire right atrium and partially invading the right ventricle. The CT showed no liver tumour or other subdiaphragmatic tumour extension. (Figure 1)

**Figure 2:**
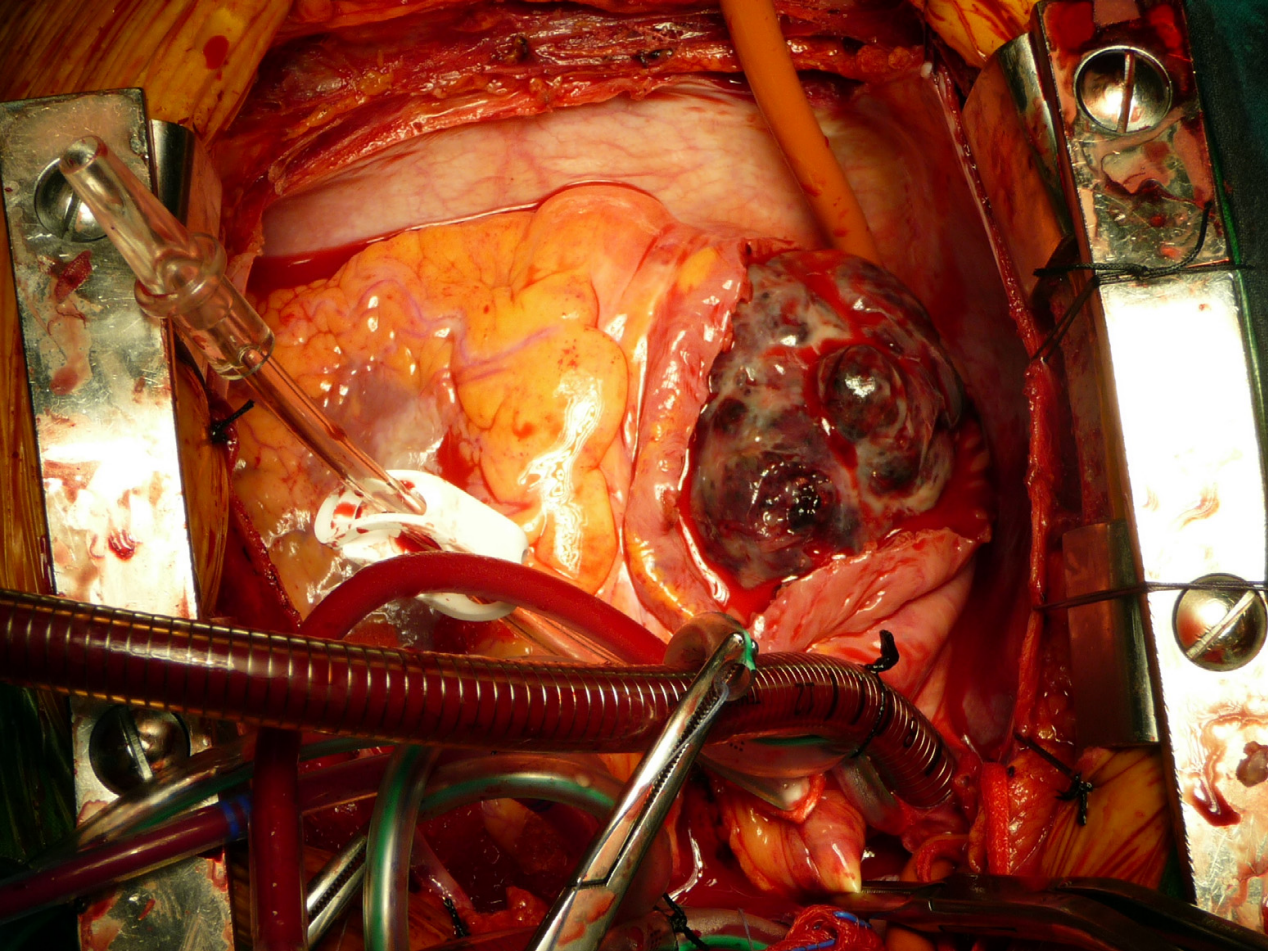
**The right atrium incised and the exposed tumor**.

**Figure 3:**
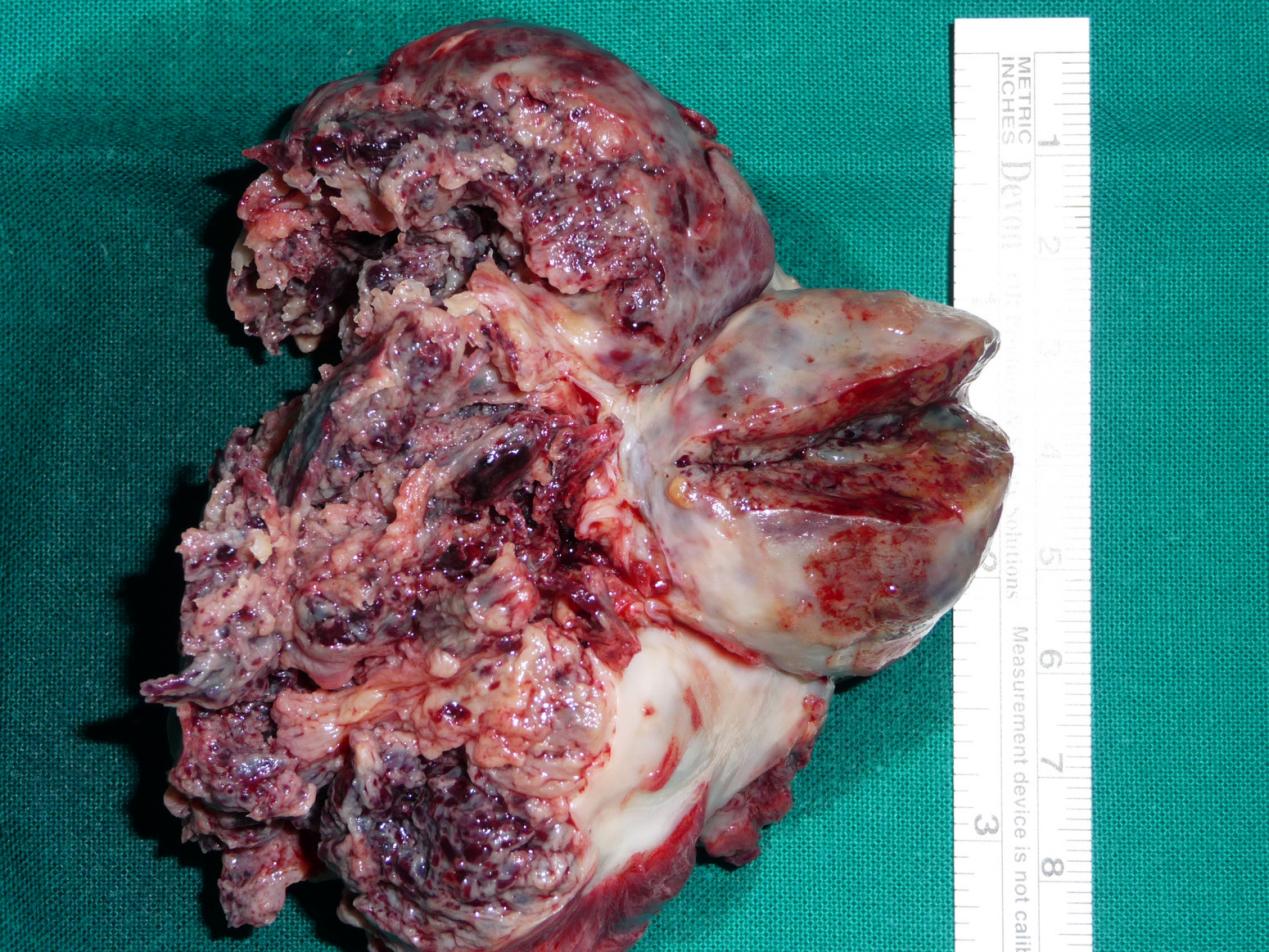
**The tumor specimen**.

The patient underwent urgent surgical treatment due to worsening of his clinical condition. The findings of the CT guided our surgical strategy as follows: Initially femoro-femoral cannulation was installed in order to commence cardiopulmonary bypass (CPB). Thus the pericardial cavity could be approached with safety. After median sternotomy, the superior vena cava was also cannulated and transfixed and then antegrade cardioplegia was administered. The heart was cooled down to 30°C, the right atrium was incised and the large tumor was carefully and copiously dissected from the surrounding tissues due to its friability. (Figure 2) The tumor originated mostly from the inferior vena cava and its terminal end was inside the right ventricle. The tricuspid valve was also invaded. The tumor was removed using a valve sparing technique. It was cautiously dissected from the tricuspid valve and the right ventricular endocardium ensuring that no remnants were left behind both on the tricuspid valvular cusps and within the vicinity of the right ventricle. The specimen was histopathologically investigated and eventually diagnosed as a metastatic hepatocellular carcinoma (HCC). (Figure 3)

Recovery was uneventful. Follow up echocardiography and cardiac MRI two months after surgery did not demonstrate any tumor recurrence or tricuspid regurgitation. Additionally PET CT and abdominal MRI showed no primary hepatoma or metastasis elsewhere in the body. No further adjuvant therapy was considered necessary in this stage by our consulting oncologists. The patient was suggested to be under closed follow-up with radiology studies (MRI, CT, and PET) in order to promptly detect solitary masses might be respectable but unfortunately he refused any other treatment option and returned to his country.

## Discussion

Primary liver cancer is the fifth most common neoplasm with an incidence of 5.5-14.9% of all tumours. Predisposing factors for orthotopic primary Hepatocellular Carcinoma (HCC) generation are chronic hepatitis B or C, infection and cirrhosis secondary to other chronic liver disease. Worldwide, the majority of patients with HCC have underlying cirrhosis, and it is uncommon to find HCC in patients without cirrhosis. Among rare cases with HCC without underlying cirrhosis, HCV infection accounted for 3-54%, HBV infection for 4-29%, and heavy alcohol intake for 0-28% [1]. Although HCC has a very aggressive metastatic profile, its tendency to spread towards the heart is unusual but well documented through several published case reports which define an incidence of cardiac metastasis at 0.67-3% [2,3]. However very few cases of giant metastatic HCC within the right cardiac cavities that cause significant occlusion of the tricuspid valve are described in the current literature.

Therefore an interesting feature of HCC can be its varied and sometimes bizarre presentation [4]. This report describes an unusual presentation of HCC. The patient appeared with symptoms of acute heart failure caused by a giant right atrial malignant obstructive hepatocellular mass without any detectable cancerous lesions in the liver. There was no radiological, clinical or laboratory suspicion of HCC. Metastatic HCC was only apparent on histology examination of the right atrial tumor. Metastatic disease as the initial presentation of HCC appears in less than 5% of cases [5]. In addition histological investigation defines whether the mass derives from an occult HCC or is presented as an ectopic one with no liver involvement.

Regarding the symptoms, there is a variety of clinical manifestations caused by the atrial neoplasm and those are mainly tumor-size dependent. Patients may have no symptoms, dyspnea due to pulmonary embolism, syncope, or heart failure. Physical findings include edema, pan systolic murmur with diastolic rumble over the tricuspid valve, and improvement of symptoms with left lateral decubitus position [6].

Extracardiac tumours involving inferior vena cava and right atrium include renal cell tumour (4-10%) [7], thyroid carcinoma, testicular tumours and HCC. In most cases of advanced HCC the extent of the disease is verified with presence of metastasis at the lungs, peritoneum, adrenal glands and bones. Generally hepatocellular carcinoma appears to have a tendency to invade vascular structures [8]. Extension to the portal vein system is common as opposed to extension into the inferior vena cava or the right atrium which is uncommon [2]. When this occurs, it mostly happens through the hepatic veins and the inferior vena cava towards the right atrium. A right atrial intracavitary mass is then formatted which causes significant hemodynamic instability. In addition, left atrium, right ventricle, and intramyocardial involvement of the left ventricle have also been reported as rare sites of HCC metastasis as well as spreading of the cancer to the left chambers through pulmonary metastasis or patent foramen ovale [9].

Regarding the case described here, the appearance of a metastatic HCC tumor inside the right atrium as the only manifestation and without apparent primary focus is unique. The chest CT angiography was the most important diagnostic modality in this case. It was very helpful in identifying the borders of the tumor. There are various sub diaphragmatic tumors including renal cell tumors and HCC which extend from below the diaphragm up to the right chambers of the heart either through the venous system or through diaphragm invasion. It is important to know the tumor location before bicaval cannulation to prevent fragmentation and embolisation of the tumor. The understanding that the tumor was well confined inside the right atrium was important for the correct planning of the procedure. CT angiogram allowed for correct placement of the arterial and venous cannulas. Thus, the arterial and one venous cannula were placed inside the femoral vessels and the utility of femoro femoral by pass circuit allowed opening of the chest with optimal safety. The other venous cannula was placed inside the superior vena cava. The right atrium was left without any cannulas in order to avoid any contact with the friable mass and minimise the risk for pulmonary embolism [10].

Intra atrial manifestation of the HCC constitutes a life threatening condition. The major causes of death are either sudden pulmonary embolism of the thrombus or acute obstruction of the tricuspid valve or both. Resection can provide relatively good mid-term survival regarding this clinical situation but not more than 2 years [11]. Standard treatment is hepatic resection with removal of the intracardial mass usually under cardiopulmonary bypass with deep hypothermia and circulatory arrest which seems to be the optimal option in most cases. A few reports describe the successful removal of HCC from the right atrium without extracorporeal circulation as an alternative [12]. However, both curative resection treatments have a dismal prognosis, with a 5 years reported survival around 12-39% [3].

After resection of a hepatocellular carcinoma, tumour recurrence exceeds up to 70% at 5 years, including recurrence due to dissemination and *de novo *tumours of the liver. The most important statistically predictor of recurrence seems to be the presence of micro vascular invasion and/or additional tumour sites besides the primary lesion [13]. There is no effective adjuvant therapy that can reduce the recurrence rates. (Recommendation level II) [13,14]

Internal radiation and adoptive immunotherapy by activated lymphocytes may have some anti-tumor efficacy but the early results have not been statistically powered as yet [15]. There are no adequate published data to indicate proper treatment of recurrences. Solitary recurrent masses might benefit from repeat resection but in the majority of cases recurrence appears to be multifocal and so further treatment is impossible [13].

## Conclusion

In conclusion, when a patient with a history of chronic hepatic disease presents with symptoms of right heart failure one must be cautious and should bear in mind that right heart involvement from a malignant tumour may be present [16]. Echocardiography computed tomography and magnetic resonance imaging are the standard imaging modalities to determine the nature of tumors presented as secondary cardiac neoplasms [16,17]. Urgent Computed tomography can easily and quickly be performed prior to surgical treatment of emergency cases. Ultrasound with liver-specific micro bubbles and PET CT can be helpful in certain cases of occult HCC [18,19].

## Consent

Written informed consent was obtained from the patient for publication of this Case report and any accompanying images. A copy of the written consent is available for review by the Editor-in-Chief of this journal.

## Competing interests

The authors declare that they have no competing interests.

## Authors' contributions

PD: Has made substantial contributions to conception and design, acquisition of data and analysis and interpretation of data. Also, has given final approval of the version to be published. IN: Has made substantial contributions to acquisition of data. IK: Has been involved in drafting the manuscript and revising it critically for important intellectual content. VA: Has made substantial contributions to acquisition of data. NP: Has made substantial contributions to acquisition of data. SZ: Has made substantial contributions to acquisition of data. EA: Has made substantial contributions to conception and design.

All authors read and approved the final manuscript.
